# A Safety-Centric Study on the Use of Inflatable Abdominal Binders for Managing Orthostatic Hypotension

**DOI:** 10.3390/clinpract14050138

**Published:** 2024-08-29

**Authors:** Milan Toma, Rejath Jose, Faiz Syed, Timothy Devine

**Affiliations:** 1 Department of Osteopathic Manipulative Medicine, College of Osteopathic Medicine, New York Institute of Technology, Old Westbury, NY 11568, USA; rjose02@nyit.edu; 2 Mather Hospital, Northwell Health, 75 N Country Rd., Port Jefferson, NY 11777, USA; fsyed09@nyit.edu; 3 The Ferrara Center for Patient Safety and Clinical Simulation, College of Osteopathic Medicine, New York Institute of Technology, Old Westbury, NY 11568, USA; tdevine@nyit.edu

**Keywords:** orthostatic hypotension, abdominal constrictor, force loads, statistical analysis

## Abstract

The study focuses on the design and evaluation of inflatable abdominal binders for managing Orthostatic Hypotension. Orthostatic hypotension is a condition characterized by a significant drop in blood pressure when a person stands up, leading to symptoms such as dizziness, lightheadedness, and even fainting. The management of orthostatic hypotension typically involves a combination of pharmacological and non-pharmacological strategies. In the context of this research, an inflatable abdominal binder was designed, leveraging components that are not only economically viable but also easily obtainable. The evaluation of this device was conducted using a medical education manikin, specifically the CAE iStan manikin. The results demonstrated a correlation between the inflation values of the belt and the resulting pressure values exerted on the body. The general recommendation for an abdominal binder is to exert a pressure of 20–40 mmHg. Contrary to this, the study found that to maintain safe external pressure on the abdomen, the binder should not be inflated over 25 mmHg. This safety threshold was used as a reference point in the study, suggesting a potential need to revisit the standard recommendations for abdominal binder pressure. Further research is needed to assess the device’s effectiveness in human subjects and to potentially redefine the safe and effective pressure range for abdominal binders.

## 1. Introduction

Orthostatic hypotension (OH) is a condition characterized by a significant drop in blood pressure upon standing, leading to symptoms such as dizziness, lightheadedness, and even syncope [1,2]. The management of OH involves a combination of pharmacological and non-pharmacological strategies [3,4]. While medications like fludrocortisone, midodrine, and droxidopa are commonly used, non-pharmacological interventions such as compression devices and lifestyle modifications play a crucial role [5]. Device-based treatments, particularly compression stockings and head-up tilt sleeping, are essential components of a comprehensive OH management plan [6].

### 1.1. Prevalence in the Elderly, Diagnostic Criteria, and Common Causes

The early recognition of OH dates back to the identification of symptoms associated with changes in body position. It has been observed across all age groups but is more prevalent in the elderly, particularly those who are frail or have underlying health conditions. Approximately 20% of older individuals, especially those in long-term care facilities, are affected by this condition [6].

The diagnosis of OH is typically based on a detailed medical history and physical examination. Healthcare providers aim to identify the cause and determine the appropriate treatment. This process involves reviewing the patient’s medical history, medications, and symptoms, and conducting a physical examination. Blood pressure monitoring is an important part of the diagnostic process, which involves measuring blood pressure while the patient is sitting and then again upon standing. A drop of 20 mm of mercury (mmHg) in systolic blood pressure within 2 to 5 min of standing is indicative of OH.

OH can be caused by various factors, including certain medications, underlying medical conditions, and neurogenic causes. Medications such as vasodilators, diuretics, antidepressants, antipsychotics, and dopaminergic drugs can commonly precipitate OH. Underlying medical conditions like diabetes, Parkinson’s disease, and cancer can also contribute to the development of OH [7].

### 1.2. Recent Case Studies and Clinical Trials

Clinical trials have shown that the binder is as effective as conventional drug therapy in controlling OH, without subjecting patients to potentially harmful side effects and interactions with other medications [8]. A single-blind crossover study investigated the efficacy of an elastic abdominal binder in controlling OH in patients with Parkinson’s disease (PD) and OH [9]. The study enrolled 15 patients who were randomly assigned to wear either an elastic abdominal binder or a placebo binder on different days. The results showed that the use of an elastic abdominal binder was associated with improved orthostatic tolerance in PD patients with OH.

A randomized crossover trial investigated the effects of patient-controlled abdominal compression on postural changes in systolic blood pressure associated with OH [10]. The study involved adults with neurogenic OH. The results suggested that mild abdominal compression prior to rising can ameliorate OH.

An automated inflatable abdominal binder has been developed as a potential treatment for OH [11]. This binder is designed to provide sustained venous compression and improve orthostatic tolerance. A randomized, double-blind, sham-controlled study is currently underway to assess the safety and efficacy of this automated abdominal binder in enhancing orthostatic tolerance. This study focuses on patients suffering from primary autonomic failure, a condition that leads to OH. The automated abdominal binder, inflated to 40 mmHg, is being compared to a sham treatment, which involves an abdominal binder inflated to just 5 mmHg. The study is projected to conclude by the end of 2024 [12].

### 1.3. Pharmacological Treatments

Fludrocortisone, a synthetic mineralocorticoid, plays a pivotal role in the management of OH by increasing extracellular fluid volume and enhancing sensitivity to catecholamines. Midodrine and Droxidopa, both vasoconstrictors, are short-acting and particularly useful during the daytime to prevent supine hypertension at night. Pyridostigmine, an acetylcholinesterase inhibitor, increases cholinergic transmission, although its effects on OH are modest and inconsistent. Atomoxetine, a selective norepinephrine transporter inhibitor, has variable effects on orthostatic blood pressure. There are also other less frequently used medications such as octreotide, erythropoietin, desmopressin, pseudoephedrine, and ergot derivatives, which are often considered as last-resort options. Combination therapies are sometimes employed to enhance treatment efficacy. For instance, fludrocortisone combined with midodrine, or ergotamine with caffeine, are used to manage OH symptoms more effectively.

### 1.4. Non-Pharmacological Interventions

Physical maneuvers such as standing with legs crossed, squatting, and active tensing of leg muscles can help mitigate the symptoms of OH by promoting venous return and stabilizing blood pressure. Raising the head of the bed by 6–9 inches can help prevent nocturnal pressure natriuresis, which is a common issue in OH patients. This simple intervention can significantly improve blood pressure stability. Increasing sodium intake (6–9 g/day) and water intake (2–3 L/day) are simple yet effective strategies to manage OH.

Compression stockings or abdominal binders with a pressure of 20–40 mmHg are recommended to reduce venous pooling in the lower extremities, thereby helping to maintain blood pressure upon standing [13,14]. However, understanding the safe external pressure values exerted on the abdominal area is crucial for preventing damage or pain, especially in clinical and therapeutic settings.

### 1.5. Safe External Pressure Values

Maintaining external pressures below 12 mmHg is advisable to avoid complications associated with elevated intra-abdominal pressure (IAP), such as impaired blood flow, reduced lung volumes, and potential organ dysfunction [15,16,17]. In healthy adults, the normal IAP ranges from 0 to 5 mmHg. This range is considered safe and does not typically cause any discomfort or damage to the abdominal organs. In critically ill patients, IAP can be slightly elevated, ranging from 5 to 7 mmHg. This elevation is generally well-tolerated and does not usually lead to significant complications. Intra-abdominal hypertension (IAH) is defined as a sustained IAP greater than 12 mmHg. At this level, there is an increased risk of complications. Abdominal compartment syndrome (ACS) is a severe condition characterized by sustained IAP greater than 20 mmHg, which can lead to end-organ damage. Bladder pressures over 25 mmHg are highly suspicious of ACS and should be correlated clinically to confirm the diagnosis and initiate appropriate treatment.

Compression devices such as abdominal binders and compression stockings are commonly used to manage conditions like OH and to support abdominal muscles post-surgery. The safe pressure exerted by these devices typically ranges from 20 to 40 mmHg [18]. Pressures within this range are generally well-tolerated and do not cause significant discomfort or damage to the abdominal area. Clinical guidelines suggest that external pressures exerted by medical devices should not exceed the thresholds that could lead to IAH or ACS [19].

## 2. Materials and Methods

This section is divided into two subsections: Design and Assessment. The Design subsection details the creation of the inflatable abdominal binder, including the materials used and their assembly. The Assessment subsection outlines the methodology used to evaluate the binder’s safety before advancing to human subjects.

### 2.1. Design

The inflatable abdominal binder was conceived by merging the inflatable segment of a sphygmomanometer cuff into a Velcro weight lifting belt. This belt, obtained from Gabor Fitness, is 39 inches long, making it adaptable to a variety of body sizes. The part of the belt where the inflatable bladder of the sphygmomanometer is affixed is 6 inches wide, ensuring a significant contact surface for efficient pressure application.

The sphygmomanometer, a product of Santa Medical, comes with an inflatable bulb and a pressure gauge. The inflatable section of the sphygmomanometer is 5 and a half inches wide, fitting perfectly within the designated area on the belt. The Velcro on both the sphygmomanometer and the belt allows for easy assembly and disassembly, providing flexibility in adjustments and maintenance.

**Figure 1:**
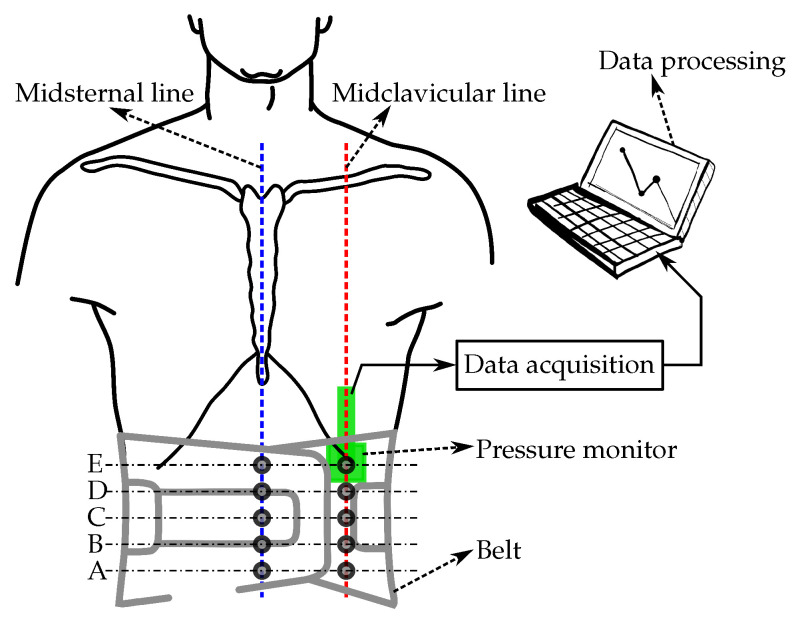
Illustration of the pressure measurement process under the inflated belt: it includes the locations along the midsternal and left midclavicular lines where the pressure values are measured, and the positioning of the belt and pressure monitor on the manikin. (Adapted from [14]).

The design enables the belt, with the attached sphygmomanometer, to be wrapped around a subject (Figure 1). The inflatable bulb is used to inflate the sphygmomanometer’s bladder to a desired external pressure. The integrated pressure gauge offers real-time feedback on the pressure inside the sphygmomanometer’s bladder, ensuring precise control of the applied pressure.

### 2.2. Assessment

The effectiveness of the inflatable abdominal binder was evaluated using a medical training manikin, specifically the CAE iStan manikin (Serial number MMPH1010) from Canadian Aviation Electronics Ltd. The iStan is a wireless manikin with capabilities such as spontaneous breathing and asynchronous mechanical ventilation, making it an excellent choice for initial data collection to verify the safety of the binder before moving on to human subjects. The manikin was arranged in a supine position with its upper body tilted at a 45-degree angle, simulating a semi-reclined human posture. The belt, equipped with a sphygmomanometer, was fastened 2–3 inches below the Xiphoid process (the lower part of the sternum) of the manikin, ensuring that the belt’s midpoint was in line with the manikin’s navel.

The JUZO pressure monitor, developed by Compression Innovations, Inc., is a device equipped with force sensors. Its purpose is to objectively measure the pressure underneath bandages and compression wraps. This device was employed to gather pressure data from various sections of the abdomen. It was positioned at five unique points, denoted as A–E, along the midclavicular and midsternal lines of the manikin’s abdomen. The central points (C) were positioned on the umbilicus and a few inches lateral to it along the midclavicular line.

The evaluation procedure included increasing the pressure in the sphygmomanometer to 20, 40, 60, and 80 mmHG levels, and noting down the corresponding pressures on the JUZO monitor at each of the points from A to E. This method was applied to both the midclavicular and midsternal lines, offering a thorough comprehension of how the pressure is distributed across the abdomen due to the binder.

## 3. Results

**Figure 2:**
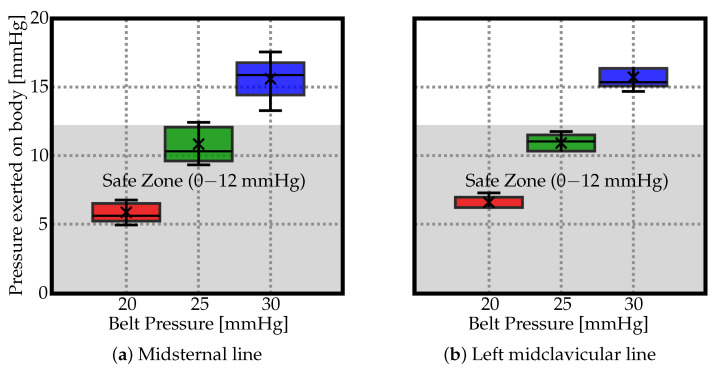
Box plots depicting groups of the data through their quartiles for (**a**) midsternal line, and (**b**) midcervical line. The ’×’ depicts the average values (see Table 1).

The correlation between the inflation values of the belt and the resulting pressure values exerted on the body is shown in Figure 2. Literature indicates that to prevent complications, the external pressure exerted on the abdomen should not exceed 12 mmHg. This safety threshold was used as a reference point in our study.

The measured pressure values are displayed in Figure 2 using box plot form depicting groups of the data through their quartiles. In the resulting charts, each rectangle was split into two parts and thin T-shaped projections on each end. The bottom end is called the local minimum. Just above it is the bottom of the box, which marks the first quartile, that is, 25th percentile. The midline inside the box represents the median. The top of the box marks the 75th percentile. The distance between the top of the box and the top end (local maximum), barring any outliers, contains the final top 25% of the measured values. No outliers were identified in the data groups.

## 4. Discussion

Clinical trials have demonstrated that an abdominal binder is as effective as conventional drug therapy in controlling OH, without the associated side effects and potential drug interactions [8]. A single-blind crossover study further supported this, showing that an elastic abdominal binder improved orthostatic tolerance in Parkinson’s disease patients with OH [9]. In a randomized crossover trial, it was found that mild abdominal compression before standing up could alleviate symptoms of OH in adults with neurogenic OH [10]. A randomized, double-blind, sham-controlled study is ongoing to evaluate the safety and efficacy of this automated abdominal binder. The study, which focuses on patients suffering from primary autonomic failure, a condition that leads to OH, is expected to conclude by the end of 2024 [12].

This study contributes to the existing body of knowledge on non-pharmacological interventions for OH. The inflatable abdominal binder designed in this study has the potential to be a practical and effective tool in the management of OH. It offers a non-invasive, adjustable, and potentially more comfortable alternative to compression stockings. The present study aimed to design and assess an inflatable abdominal binder for the management of OH. The device was designed by integrating the inflatable portion of a sphygmomanometer cuff into a velcro weight lifting belt. The assessment was conducted using a medical education manikin, the CAE iStan manikin.

The results demonstrated a correlation between the inflation values of the belt and the resulting pressure values exerted on the body. This finding is applicable as it provides a quantifiable measure of the pressure exerted by the device, which can be controlled and adjusted according to the needs of the individual user.

According to literature, to prevent complications, the external pressure exerted on the abdomen should not exceed 12 mmHg. This safety threshold was used as a reference point in this study, and it was found that an inflation parameter for the belt should not exceed 25 mmHg to maintain external pressures beneath this threshold.

Hence, while the commonly suggested range of pressure values with which abdominal binders should be inflated is 20–40 mmHg, our study found that values over 25 mmHg may potentially pose safety concerns. This suggests a need for further research and possibly a reevaluation of the recommended pressure values for abdominal binders. Further research is needed to assess the device’s effectiveness in human subjects and to further explore the implications of our findings on the recommended pressure values for abdominal binders.

While our study provides preliminary data on the use of inflatable abdominal binders for managing OH, it is important to consider the limitations when interpreting our findings. Our study utilized a manikin instead of a living patient. While this approach allowed us to control for numerous variables and focus on the external pressures exerted by the binder, it does not account for the individual physiological responses that might occur in a living patient. Therefore, the results may not fully represent the effects of the binder in a clinical setting. Moreover, the use of a manikin limits our ability to assess the comfort and tolerability of the binder, which are important factors in patient compliance with treatment. Future studies involving living patients would be beneficial to evaluate these aspects.

## 5. Conclusions

Our findings challenge the previously accepted safe pressure inflation range of 20–40 mmHg for abdominal binders. Our data suggests a more conservative upper limit of 25 mmHg to ensure the intra-abdominal pressure remains below 12. This adjustment is not only important for patient safety but also for comfort, while still managing orthostatic hypotension. The results provide a more refined understanding of how to balance the efficacy of abdominal binders with patient safety. Though, further research is necessary to confirm these results and to better understand their implications.

## Figures and Tables

**Table 1:** The resultant mean pressure readings (mmHg), measured along the midsternal and midclavicular lines corresponding to each specified inflation pressure of the belt. Given that it is recommended to maintain external pressures below the threshold of 12 mmHg (highlighted in gray rows) to circumvent adverse outcomes, an inflation parameter for the belt should not exceed 25 mmHg.

	Mean Pressure Exerted on	
**Belt Inflated by:**	**Midsternal Line**	**Midclavicular Line**
20 mmHg	5.6	6.8
25 mmHg	11.65	11.5
30 mmHg	16.3	16.7

## Data Availability

The original contributions presented in the study are included in the article, further inquiries can be directed to the corresponding author.

## Abbreviations

The following abbreviations are used in this manuscript:

OH	orthostatic hypotension
mmHG	millimeters of mercury
PD	Parkinson’s disease
IAP	intra-abdominal pressure
IAH	intra-abdominal hypertension
ACS	abdominal compartment syndrome
